# A predictive model, and predictors of under-five child malaria prevalence in Ghana: How do LASSO, Ridge and Elastic net regression approaches compare?

**DOI:** 10.1016/j.pmedr.2021.101475

**Published:** 2021-06-27

**Authors:** Justice Moses K. Aheto, Henry Ofori Duah, Pascal Agbadi, Emmanuel Kweku Nakua

**Affiliations:** a Department of Biostatistics, School of Public Health, College of Health Sciences, University of Ghana, Ghana; b WorldPop, University of Southampton, United Kingdom; c Research Department, FOCOS Orthopaedic Hospital, Accra, Ghana; d Department of Nursing, Faculty of Allied Health Sciences, College of Health Sciences, Kwame Nkrumah University of Science and Technology, Kumasi, Ghana; e Department of Epidemiology and Biostatistics, School of Public Health, Kwame Nkrumah University of Science and Technology, Kumasi, Ghana

**Keywords:** LASSO, RIDGE, Elastic net, Malaria, Ghana

## Abstract

Malaria is among the leading causes of mortality and morbidity among children in Ghana. Therefore, identifying the predictors of malaria prevalence in children under-five is among the priorities of the global health agenda. In Ghana, the paradigm shifts from using traditional statistics to machine learning techniques to identifying predictors of malaria prevalence are scarce. Thus, the present study used machine learning techniques to identify variables to build the best fitting predictive model of malaria prevalence in Ghana. We analysed the data on 2867 under-five children with malaria RDT results from the 2019 Ghana Malaria Indicator Survey. LASSO, Ridge, and Elastic Net regression methods were used to select variables to build predictive models. The R freeware version 4.0.2 was used. One out of four children tested positive for malaria (25.04%). The logit models based on selected features by LASSO, Ridge, and Elastic Net contained eleven, fifteen, and thirteen features, respectively. The LASSO regression model is preferred because it contains the smallest number of predictors and the smallest prediction error. The significant predictors of malaria among children were being older than 24 months, residing in the poorest household, being severely anaemic, residing in households without electricity, and residing in a rural area. The predictors identified in our study deserve policy attention and interventions to strengthen malaria control efforts in Ghana. The machine learning techniques employed in our study, especially the LASSO regression technique could be beneficial for identifying predictors of malaria prevalence in this group of children.

## Highlights

• One out of four under-five children (U5C) tested positive for malaria in Ghana.
• U5C older than 24 months or those severely anaemic were more likely to test positive for malaria.
• U5C residing in households without electricity were more likely to test positive for malaria.
• U5C residing in a rural area were more likely to test positive for malaria.
• U5C residing in the poorest household were more likely to test positive for malaria.

## 1 Introduction

Globally, childhood malaria remains one of the leading causes of under-five morbidity and mortality in Sub-Saharan Africa (SSA) (Maitland, 2016, Camponovo et al., 2017). Malaria is known to cause haemolysis of red blood cells (erythrocytes) coupled with the formation of abnormal red blood cells (dyserythropoietic) all of which culminate in the development of anaemia in children (White, 2018). Complications of malaria have unfavourable clinical outcomes with significant case-fatality rate (Aponte et al., 1999). Therefore, childhood malaria has been taken seriously by clinicians and policymakers over the years.

Substantial global policy initiatives have been implemented since the early 2000s to curb the burden of malaria in SSA. An example is the United States President’s Malaria Initiative (PMI) which was launched in 2005 and consequently led to an increased availability of insecticide-treated mosquito nets (ITNs), antimalarial treatments and rapid diagnostic tests and indoor residual spraying amongst others. The PMI has led to a significant reduction in under-five mortality in SSA (Jakubowski et al., 2017). Following the success of the “*for a malaria-free world 2008*–*2015* initiative”, the Roll Back Malaria Partnership outlined an action plan in dubbed, “Action and Investment to Defeat Malaria (AIM) 2016–2030” (Partnership and Action, 2015). The alignment of the timeframe of the vision of AIM to that of the Sustainable Development Goals (SDG) underscores the need to address the problem of under-five malaria to ensure the realization of SDG goal 3. Nevertheless, malaria continues to be a significant cause of childhood deaths in SSA, thus threatening to derail the gains towards the achievement of the sustainable development Goal 3.2 which seeks to reduce under-5 mortalities to at least as low as 25 per 1000 live births by 2030.

The potential adverse outcome after childhood malaria underscores the need for early detection and identification of high-risk populations. Researchers have over the years used a variety of predictive modelling approaches to identify high-risk populations. These have included correlation studies, standard linear and logistic regression models, Poisson regression, non-linear models, an autoregressive integrated moving average models (ARIMAs) and spatial mapping approaches (Zhou et al., 2004, Wangdi et al., 2010, Bi et al., 2003, Craig et al., 2004, Weiss et al., 2019, Millar et al., 2018, Yankson et al., 2019). These predictive modelling approaches are largely limited by the number of covariates that can be fitted and are usually subject to the intuition of the researcher. For conditions such as malaria which is influenced by a range of physical, climatic, and social factors, machine learning models provide the opportunity to fit many covariates to identify high-risk populations. Wang et al. (Wang et al., 2019) demonstrated the superiority in the use of ensemble algorithms in predicting malaria in China using secondary health data. However, there is a paucity of literature in the Ghanaian context utilizing machine learning algorithms to predict malaria in children under five. This study sought to fill the gap in the literature by using LASSO, ridge, and Elastic net regression models to build a predictive model of malaria prevalence in children under five years in Ghana.

## 2 Materials and data

### 2.1 Design, data collection, and study sample

We analyzed the data on children under-five from the 2019 Ghana Malaria Indicator Survey (GMIS) (Ghana Statistical Service (GSS), 2019). The GMIS is based on a two-stage sampling design. The sampling was based on ten administrative regions. Each region was divided into urban and rural areas, resulting in twenty sampling strata. Enumeration areas (EAs) were sampled from each stratum. In the first stage, 200 EAs (97 in urban areas and 103 in rural areas) were selected with probability proportional to EA size (Ghana Statistical Service (GSS), 2019). In the second stage of selection, a fixed number of 30 households were selected from each cluster to make up a total sample size of 6,000 households (Ghana Statistical Service (GSS), 2019). About 5,181 women age 15–49 (representing 98.8% response rate) who were either permanent residents of the selected households or visitors who stayed in the household the night before the survey were eligible to be interviewed (Ghana Statistical Service (GSS), 2019). With the parent’s or guardian’s consent, children age 6–59 months were tested for anaemia and malaria infection (Ghana Statistical Service (GSS), 2019). The biomarker dataset has malaria RDT results on 2867 children under-five in Ghana.

## 3 Measures

### 3.1 Outcome variable

The outcome variable is children who tested positive for malaria through a rapid diagnostic test (RDT) kit. The RDT malaria test for children under-five was conducted by taking a drop of blood with the SD BIOLINE Malaria Ag P.f rapid diagnostic test (RDT). This test kit produces a result in 15 min (Ghana Statistical Service (GSS), 2019). The SD BIOLINE P.f RDT tests for one antigen, histidine-rich protein II (HRP-II), specific to Plasmodium falciparum (Pf), the major cause of malaria in Ghana (Ghana Statistical Service (GSS), 2019).

### 3.2 Explanatory variables

The selection of explanatory variables was informed by literature search and their availability in the dataset. These variables include the following: child age, number of under-five children in a household, has mosquito bed net for sleeping, sex of household head, sex of a household member, dwelling sprayed against mosquito last 12 months, household wealth, sex of household head, child-anaemia status, has electricity in HH, has a television in the household, place of residence, the region of residence, number of children who slept under mosquito bed net previous night, insecticide-treated net available in the household, number of household members.

### 3.3 Statistical analyses

We describe the characteristics of the study sample by using frequency and percentages. Chi-square test of independence was performed between the outcome and the explanatory variables. We used the Least Absolute Shrinkage and Selection Operator (LASSO), Ridge, and Elastic Net regression methods to identify variables to build the best fitting predictive model of malaria prevalence in Ghana. For LASSO, an alpha value of one was used and for Ridge an alpha value of 0. Given that the alpha values for Elastic net lie between an alpha value of zero and one (i.e. 0 < alpha < 1), we performed maximum likelihood to obtain the alpha value which was estimated to be 0.4186508 based on 5-fold cross-validation, repeated five times using ‘caret’ package. We estimated the minimum lambda (i.e., lowest mean squared error (MSE)) for LASSO, Ridge and Elastic net via maximum likelihood estimation under k-fold cross-validation. The ‘glmnet’ package was used to select features for all models under the machine learning approaches.

Let Y be the malaria indicator. We set the binary response $Y_{i}=\left\{\begin{array}{ll} 1 & \mathrm{if}\;\mathrm{the}\;i-th\;child\;had\;malaria\\ 0 & \mathrm{otherwise} \end{array}\right)$ and assume $\pi_{i}$ to be the probability that a given child *i* had malaria. Thus, our model formulation for the multivariable binary logistic regression for predicting under-five malaria status is: $log\left(\frac{\pi_{i}}{1-\pi_{i}}\right)=\beta_{0}+d{\left(x_{ijk}\right)}^{\text{'}}\beta$, where $\beta_{0}$ is the intercept, $d\left(.\right)$ is a vector of predictors and β is a vector of regression coefficients for the predictors in the model. We extend this model to incorporate the regularization parameters for LASSO, Ridge and Elastic net models.

After fitting the model to the full dataset, we split the data into 80% and 20% training and validation sets respectively. We then fit models to these data and evaluate their predictive ability via AUC Curves. To examine any evidence of multicollinearity, we employed the generalized variance inflation factor (GVIF) (Hair et al., 2018, Fox and Monette, 1992) with a GVIF value below 10 considered acceptable (Hair et al., 2018). The goodness of fit of the model was tested using Hosmer and Lemeshow goodness of fit (GOF) test. The fit was also examined using McFadden's R^2^, and a model with a value between 0.2 and 0.4 is considered an excellent fit. All analyses were performed in the R freeware version 4.0.2 (Core-Team R, 2019).

### 3.4 Ethical consideration

We obtained permission to use the 2019 GMIS data from the DHS MEASURE Program which is freely available after a simple, registration-access request at the following address https://dhsprogram.com/data/dataset_admin/index.cfm. From their report, it is indicated that the protocol for the 2019 GMIS was approved by the Ghana Health Service Ethical Review Committee and ICF’s Institutional Review Board (Ghana Statistical Service (GSS), 2019).

## 4 Results

In the sample, one out of four children tested positive for malaria (25.04%) (see Table 1). The factors that were significantly associated with malaria among children include child age, number of under-five children, has mosquito bed net for sleeping, under-five children who slept under mosquito bed net last night, sex of household, Household wealth, Anaemia level, has electricity in household, has Television in the household, place of residence, the region of residence, number of children who slept under mosquito bed net previous night, insecticide-treated net, and number of household members  (see Table 1).

**Table 1:** Descriptive statistics.

Study variables	N (%)	N (%)	
**Malaria Prevalence**			
Negative (-VE)	2149 (74.96%)		
Positive (+VE)	718 (25.04)		
Total	2867		
	**Malaria Prevalence**		
	-VE	+VE	
**Child Age (in months)**			
< 24 months	769 (82.51)	163 (17.49)	χ^2^ = 46.05, p ≤ 0.001
24–48 months	972 (72.65)	366 (27.35)	
> 48 months	408 (68.34)	189 (31.66)	
**Number of U5C in household**			
0–1	910 (77.65)	265 (22.35)	χ^2^ = 25.04, p ≤ 0.005
2–3	1085 (73.81)	385 (26.19)	
> 3	154 (68.44)	71 (31.56)	
**Has mosquito bed net for sleeping**			
No	312 (80.0)	78 (20.00)	χ^2^ = 6.12, p ≤ 0.05
Yes	1837 (74.16)	640 (25.84)	
**U5C slept under mosquito bed net last night**			
no	546 (82.98)	112 (17.02)	χ^2^ = 43.23, p ≤ 0.001
all children	1044 (70.92)	428 (29.08)	
some children	247 (71.18)	247 (28.82)	
no net in household	312 (80.00)	78 (20.00)	
**Sex of HH**			
Male	1533 (74.74)	518 (25.26)	χ^2^ = 0.17, p = 0.68
Female	616 (75.49)	200 (24.51)	
**Sex of household member**			
male	1081 (73.84)	383 (26.16)	χ^2^ = 1.99, p = 0.16
Female	1068 (76.12)	335 (23.88)	
**Dwelling sprayed against mosquito last 12 months**			
no	1758 (74.40)	605 (25.60)	χ^2^ = 2.24, p = 0.13
Yes	391 (77.58)	113 (22.42)	
**Household wealth**			
Poorest	619 (64.48)	341 (35.52)	χ^2^ = 214.24, p ≤ 0.001
Poorer	420 (67.42)	203 (32.58)	
Middle	431 (77.38)	126 (22.62)	
Richer	369 (90.00)	41 (10)	
Richest	310 (97.79)	7 (2.21)	
**Anaemia level**			
Severe	10 (20.41)	39 (79.59)	χ^2^ = 267.76, p ≤ 0.001
Moderate	504 (59.57)	342 (40.43)	
Mild	603 (77.91)	171 (22.09)	
Not anaemic	1032 (86.14)	166 (13.86)	
**Has electricity in HH**			
No	475 (59.45)	324 (40.55)	χ^2^ = 141.90, p ≤ 0.001
Yes	1674 (80.95)	394 (19.05)	
**Has Television in HH**			
No	822 (65.29)	437 (34.71)	χ^2^ = 111.74, p ≤ 0.001
Yes	1327 (82.52)	281 (17.48)	
**Place of residence**			
Urban	944 (89.06)	116 (10.94)	χ^2^ = 178.12, p ≤ 0.001
Rural	1205 (66.69)	602 (33.31)	
**Region of residence**			
Western	205 (71.18)	83 (28.82)	χ^2^ = 123.90, p ≤ 0.001
Central	174 (67.97)	82 (32.03)	
Greater Accra	184 (98.92)	2 (1.08)	
Volta	172 (68.53)	79 (31.47)	
Eastern	160 (71.11)	65 (28.89)	
Ashanti	240 (85.71)	40 (14.29)	
Brong Ahafo	165 (62.03)	101 (37.97)	
Northern	406 (79.45)	105 (20.55)	
Upper East	210 (70.00)	90 (30.00)	
Upper West	233 (76.64)	71 (23.36)	
**Number of children who slept under mosquito bed net previous night**			
No child	858 (81.87)	190 (18.13)	χ^2^ = 42.48, p ≤ 0.001
1–2 children	1157 (71.20)	468 (28.80)	
>3 children	134 (69.07)	60 (30.93)	
**Insecticide-treated net**			
No	973 (80.28)	239 (19.72)	χ^2^ = 31.70, p ≤ 0.001
Yes	1176 (71.06)	479 (28.94)	
**number of household members**			χ^2^ = 17.03, p ≤ 0.001
< 6 members	1038 (78.46)	285 (21.54)	
Members	822 (72.61)	310 (27.39)	
> 9 members	289 (70.15)	123 (29.85)	

### 4.1 Feature selection to build the predictors of malaria prevalence model

**Fig. 1:**
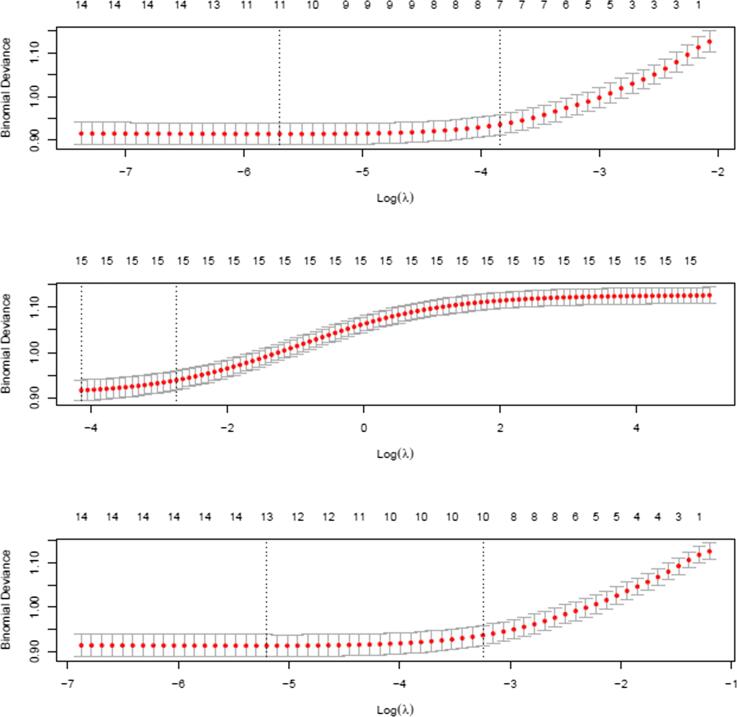
The binomial deviance versus the log(Lambda) plots. Note: 1st row: LASSO; 2nd row: RIDGE; 3rd row: ELASTIC NET.

LASSO, Ridge, and Elastic Net regressions were used for feature selection to build a predictive model of malaria prevalence (Table 2), and the binomial deviance versus the log(Lambda) plots are presented as Fig. 1. The variables included in each of the feature selection models were: child age, number of under-five children in a household, has mosquito bed net for sleeping, sex of household head, sex of a household member, dwelling sprayed against mosquito last 12 months, household wealth, sex of household head, child-anaemia status, has electricity in HH, has a television in the household, place of residence, the region of residence, number of children who slept under mosquito bed net previous night, insecticide-treated net available in the household, number of household members. Per the LASSO results, the best fitting models excluded these variables: has mosquito bed net for sleeping, sex of a household member, and the number of household members. The ridge regression results included all the fifteen features. For the Elastic Net regression results excluded these two variables: has mosquito bed net for sleeping and sex of household member.

**Table 2:** Lasso, Ridge, and Elastic Net.

	LASSO	RIDGE	ELASTIC NET
	alpha = 1	alpha = 0	alpha = 0.4186508
(Intercept)	1.043406015	0.81622002	0.957635393
Region	−0.125009418	−0.11751371	−0.125348706
Urban-rural residence	0.797182998	0.79393783	0.806272194
Has electricity in HH	−0.334558348	−0.35767631	−0.353889442
Has Television in HH	.	−0.06166143	−0.001048464
Sex of HH	0.019345178	0.07802045	0.047635449
Has mosquito bed net for sleeping	.	−0.08209747	.
Household wealth index	−0.356708586	−0.31547715	−0.352298541
sex of household member	.	−0.02806722	.
Anaemia level	−0.821035391	−0.77787099	−0.817498792
Dwelling sprayed against mosquito last 12 months	−0.350619294	−0.38498364	−0.367712726
Number of children who slept under mosquito bed net previous night	0.022397129	0.05179602	0.025362654
Number of U5C in household	0.003430091	0.03509552	0.02308751
Insecticide-treated net	0.136570517	0.18949137	0.157816524
Child Age	0.651001735	0.619984	0.652697693
number of household members	.	0.02590606	0.000677887

The plot in Fig. 1 displays the cross-validation error according to the log of the regularization parameter (lambda). The left dashed black vertical line indicates the optimal value of lambda which is the one that minimizes the prediction error (i.e., binomial deviance). This lambda value is expected to provide the most accurate model. For example, the top plot in Fig. 1 indicates that log of lambda of approximately −5.7 will be the one that minimizes the prediction error with 11 features selected.

### 4.2 Predictive ability of the feature selected models of LASSO, RIDGE, and Elastic Net

**Fig. 2:**
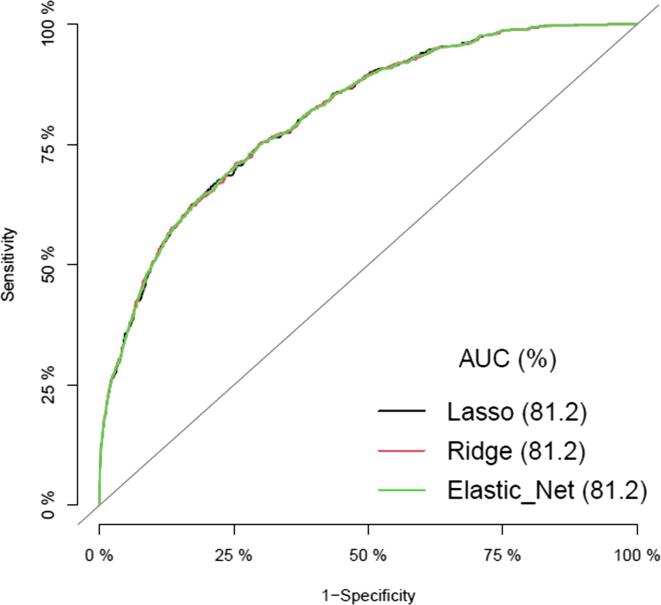
Area under the curve values for the LASSO, RIDGE, AND Elastic Net feature selected models.

We build three logit models, each with the features selected by LASSO, RIDGE, and Elastic Net regressions. The logit models based on selected features by LASSO, RIDGE, and Elastic Net contained eleven features, fifteen features, and thirteen features, respectively. All the models explained about 20% of the variability in malaria prevalence in Ghana with the same area under the curve (AUROC) values (i.e., AU = 81.20%) indicating that the models were good at predicting malaria prevalence in this group of children (Table 3, Fig. 2). Based on the principle of parsimony, the Lasso regression is preferred because it contains the smallest number of predictors and the smallest prediction error. We also presented the root mean square error (RMSE, i.e., prediction error) as a performance indicator for our models based on the cross-validation estimates obtained. The best model is the one with the lowest predictive error. Here again, the LASSO model (RMSE = 0.9489, SD = 0.0202) performed relatively better than the Ridge (RMSE = 1.0366, SD = 0.0172) and Elastic net (RMSE = 0.9531, SD = 0.0190) models (Table 3), supporting the choice of the LASSO model. Thus, only the results in the LASSO selected feature logit model was interpreted.

**Table 3:** Explained variance and area under the curve results for LASSO, RIDGE, and Elastic Net.

Model	R-Square	RMSE (95% CI)	SD	AUC Value	Number
Lasso	0.196989	0.9489 (0.9286, 0.9691)	0.0202	81.20%	11
Ridge	0.1972966	1.0366 (1.0194, 1.0537)	0.0172	81.20%	15
Elastic net	0.1971316	0.9531 (0.9342, 0.9721)	0.0190	81.20%	13

We further examined our final (i.e., LASSO) model to detect any presence of multicollinearity using the generalized variance inflation factor (GVIF). All the estimates of GVIFs are below 3, suggesting that there is no evidence of multicollinearity. The Hosmer and Lemeshow goodness of fit (GOF) test reveals no evidence of lack of fit (${x^{2}}_{8}$ = 13.6, p-value = 0.0939). Also, the McFadden's R^2^ of 0.23 revealed an excellent fit for our final model.

### 4.3 Evaluation of model fit on training and validation datasets

**Fig. 3:**
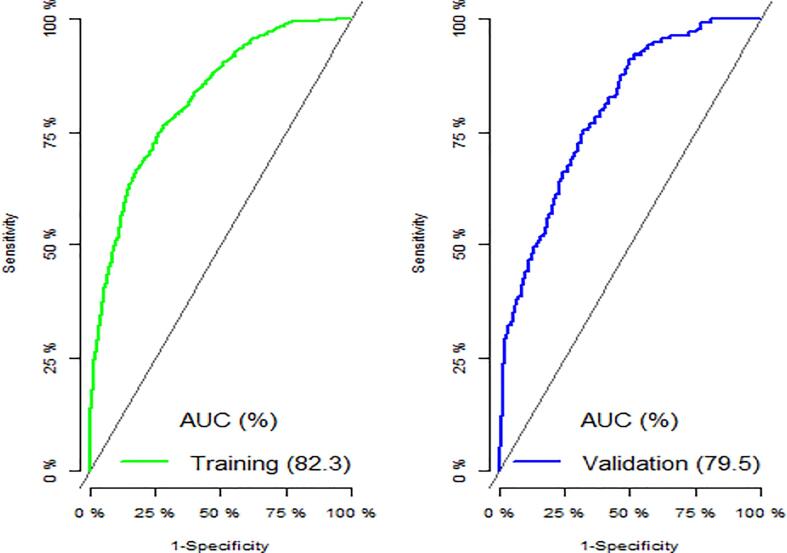
Area under the ROC curve comparing the predictive ability of the training and the validation sets.

We tested our final preferred model (i.e., LASSO) on the training dataset using Hosmer and Lemeshow goodness of fit (GOF) test. We did not observe any evidence of lack of fit (${x^{2}}_{8}$ = 11.8, p-value = 0.1627). Also, the McFadden's R^2^ of 0.25 and 0.21 respectively for the training and validation dataset models indicate an excellent fit for our model. The predictive ability of the fitted model based on AUC values for the training and validation datasets are respectively 82.3% and 79.5% (Fig. 3), indicating good predictive ability for both. We test for any difference in the predictive performance between the fitted model for the training and the validation sets by comparing the ROC curves for these models. Both the DeLong's (D = 1.1993, p-value = 0.2308) and Bootstrap (D = 1.197, p-value = 0.2313) tests for the two ROC curves suggest that there is no evidence of significant differences in the predictive performance of these models.

### 4.4 Regressors of malaria prevalence in Ghana

The following factors were regressed upon malaria prevalence in Ghana: child age, number of under-five children in a household, sex of household head, dwelling sprayed against mosquito last 12 months, household wealth, child-anaemia status, has electricity in households, place of residence, the region of residence, number of children who slept under mosquito bed net previous night, and insecticide-treated net available in the household.

The factors that are significantly related to the outcome were child age, household wealth, child anaemia status, presence of electricity in household, place of residence, and region of residence. The adjusted odds ratios reported in Table 4 are reported as follows. Compared to children who are less than 24 months, children who are 24–48 months old [AOR = 2.63, 95% CI: 2.06, 3.36] and more than 48 months old [AOR = 4.28, 95% CI: 3.19, 5.77] were more likely to test positive for malaria. Compared to children in the poorest households, children in the middle [AOR = 0.59, 95% CI: 0.40, 0.86], richer [AOR = 0.33, 95% CI: 0.20, 0.52], and richest [AOR = 0.10, 95% CI: 0.04, 0.23] households were less likely to test positive for malaria. Compared to children with severe anaemia status, children with moderate [AOR = 0.16, 95% CI: 0.07, 0.34], mild [AOR = 0.05, 95% CI: 0.02, 0.11], and not anaemic [AOR = 0.03, 95% CI: 0.01, 0.06] were less likely to test positive for malaria. Compared to children in households without electricity, children in households with electricity [AOR = 0.68, 95% CI: 0.53, 0.87] were less likely to test positive for malaria. Compared to children in urban areas [AOR = 2.09, 95% CI: 1.58, 2.77], children in rural areas were more likely to test positive for malaria. Compared to children in Western region, children in Greater Accra [AOR = 0.08, 95% CI: 0.01, 0.29], Ashanti [AOR = 0.49, 95% CI: 0.30, 0.79], Northern [AOR = 0.17, 95% CI: 0.11, 0.26], Upper East [AOR = 0.37, 95% CI: 0.23, 0.57], and Upper West [AOR = 0.31, 95% CI: 0.18, 0.54] were less likely to test positive for malaria.

**Table 4:** Regressors of Malaria Prevalence in Ghana.

	LASSO	RIDGE	ELASTIC NET
Variables	**AOR [95% CI]**	**AOR [95% CI]**	**AOR [95% CI]**
(Intercept)	4.35 [1.81, 11.10]	4.76 [1.92, 12.56]	4.33 [1.79, 11.12]
**Child Age**			
< 24 months	1	1	1
24–48 months	2.63 [2.06, 3.36]	2.61 [2.05, 3.34]	2.61 [2.05, 3.35]
> 48 months	4.28 [3.19, 5.77]	4.26 [3.18, 5.74]	4.26 [3.18, 5.75]
**Number of U5C in household**			
0–1	1	1	1
2–3	1.12 [0.90, 1.39]	1.09 [0.87, 1.36]	1.09 [0.87, 1.36]
> 3	1.54 [0.99, 2.38]	1.33 [0.81, 2.18]	1.34 [0.81, 2.19]
**Sex of HH**			
Male	1	1	1
Female	0.97 [0.77, 1.22]	0.97 [0.77, 1.23]	0.98 [0.77, 1.23]
**Dwelling sprayed against mosquito last 12 months**			
no	1	1	1
Yes	0.83 [0.57, 1.20]	0.83 [0.57, 1.20]	0.83 [0.57, 1.20]
**Household wealth**			
Poorest	1		
Poorer	0.81 [0.61, 1.09]	0.82 [0.61, 1.11]	0.82 [0.61, 1.10]
Middle	0.59 [0.40, 0.86]	0.60 [0.40, 0.90]	0.60 [0.40, 0.90]
Richer	0.33 [0.20, 0.52]	0.34 [0.20, 0.56]	0.33 [0.20, 0.56]
Richest	0.10 [0.04, 0.23]	0.11 [0.04, 0.24]	0.11 [0.04, 0.24]
**Anaemia level**			
Severe	1		
Moderate	0.16 [0.07, 0.34]	0.16 [0.07, 0.34]	0.16 [0.07, 0.33]
Mild	0.05 [0.02, 0.11]	0.05 [0.02, 0.11]	0.05 [0.02, 0.11]
not anaemic	0.03 [0.01, 0.06]	0.03 [0.01, 0.06]	0.03 [0.01, 0.06]
**Has electricity in HH**			
No	1		
Yes	0.68 [0.53, 0.87]	0.68 [0.52, 0.90]	0.68 [0.52, 0.89]
**Place of residence**			
Urban	1		
Rural	2.09 [1.58, 2.77]	2.09 [1.58, 2.77]	2.08 [1.57, 2.76]
**Region of residence**			
Western	1	1	1
Central	0.90 [0.58, 1.38]	0.90 [0.58, 1.38]	0.90 [0.58, 1.38]
Greater Accra	0.08 [0.01, 0.29]	0.08 [0.01, 0.28]	0.08 [0.01, 0.28]
Volta	0.72 [0.47, 1.11]	0.72 [0.47, 1.10]	0.72 [0.47, 1.10]
Eastern	0.94 [0.60, 1.46]	0.95 [0.61, 1.47]	0.94 [0.60, 1.47]
Ashanti	0.49 [0.30, 0.79]	0.49 [0.30, 0.78]	0.49 [0.30, 0.78]
Brong Ahafo	0.81 [0.53, 1.23]	0.80 [0.53, 1.22]	0.80 [0.53, 1.22]
Northern	0.17 [0.11, 0.26]	0.16 [0.10, 0.26]	0.16 [0.10, 0.26]
Upper East	0.37 [0.23, 0.57]	0.36 [0.23, 0.56]	0.36 [0.23, 0.56]
Upper West	0.31 [0.18, 0.54]	0.30 [0.17, 0.52]	0.30 [0.17, 0.52]
**Number of children who slept under mosquito bed net previous night**			
No child	1	1	1
1–2 children	1.15 [0.75, 1.76]	1.18 [0.75, 1.86]	1.12 [0.72, 1.72]
>3 children	1.02 [0.55, 1.85]	1.04 [0.56, 1.94]	0.98 [0.53, 1.79]
**Insecticide-treated net**			
No	1	1	1
Yes	1.04 [0.69, 1.56]	1.08 [0.72, 1.63]	1.08 [0.74, 1.63]
**Has Television in HH**			
no	—	1	1
yes	—	0.97 [0.74, 1.28]	0.98 [0.74, 1.29]
**number of household members**	—		
< 6 members	—	1	1
6–9 members	—	1.04 [0.83, 1.31]	1.04 [0.83, 1.31]
> 9 members	—	1.25 [0.86, 1.79]	1.25 [0.86, 1.79]
**sex of household member**	—		—
male	—	1	—
female	—	0.95 [0.78, 1.16]	—
**Has mosquito bed net for sleeping**	—		—
No	—	1	—
Yes	—	0.87 [0.59, 1.28]	—

## 5 Discussion

This study finds that in 2019, one out of four children tested positive for malaria (25.04%) with considerable malaria prevalence across different age group of children under five years. Our results also showed a good predictive ability of our fitted models (i.e., AU = 81.20%) to predict under-five malaria prevalence. Factors that were significantly associated with malaria prevalence in Ghana included: child age, household wealth, child anaemia status, presence of electricity in household, place of residence, and region of residence.

We found that children older than 24 months were more likely to test positive for malaria. This finding may be attributable to multiple reasons. One plausible explanation is the age-related decline in malaria antibodies acquired from the mother during pregnancy as the child grows. Although there is no consensus in the literature on the effect of maternally acquired immunity in protecting against childhood malaria (Riley et al., 2001, Riley et al., 1998), the assumption is that children in malaria-endemic areas such as Ghana acquire malaria antibodies from their mothers while in the womb but this immunity wanes gradually as the child grows. This coupled with low utilization of insecticide-treated nets in children older than 24 months (Nkoka et al., 2019) due to prioritization of access to ITN for younger siblings may explain our observation that older than 24 months are more likely to test positive for malaria. Moreover, mothers with an index child much younger are given more attention than those 24 months or more, hence the latter are more likely to be exposed to mosquito bites. Other studies have also reported a significant association between age and malaria infections in children in Ghana (Nyarko and Cobblah, 2014, Orish et al., 2015, Chilanga et al., 2020). In the retrospective study in the Western Regional Hospital in Ghana, Orish *et al* (Orish et al., 2015), noted that the age-specific discrepancy in the prevalence of malaria was rather higher for younger children. This variance is understandable given that although community prevalence of malaria may be actually higher for older children, the health seeking behaviours of parents plausibly prioritise younger children with malaria for treatment.

We also found that children in at least a middle wealthy household had a lower likelihood of testing positive for malaria. This finding can be explained by the fact that children from wealthy households are more likely to be living in affluent neighbourhoods with good drainage system and clean environments that decrease the breeding of mosquitoes thus decreasing the likelihood of mosquito bites and malaria (Dickinson et al., 2012). Moreover, parents/guardians of children from wealthy households are more likely to afford the purchase and use of ITN (Dickinson et al., 2012, Ruyange et al., 2016) hence reducing the likelihood of malaria in children from wealthy families. This finding corroborates the findings of other studies in Ghana (Nyarko and Cobblah, 2014, Afoakwah et al., 2018) and other African countries (West et al., 2013) which all reported lower burden of malaria among children from wealthy households.

The study found that children who were not severely anaemic and not anaemic at all had a lower likelihood of testing positive for malaria. The association between anaemia and malaria in SSA has been well documented in the literature (McCuskee et al., 2014). This finding can be related to the haemolytic effect of malaria on red blood cells causing anaemia (White, 2018). This likely explains why mildly anaemia and non-anaemic children were less likely to test positive for malaria.

The study also found that children in households with electricity had a lower likelihood of testing positive for malaria. Access to electricity can be understood as a proxy for wealth status, access to other social amenities and socioeconomic status (Worrall et al., 2003). The assumption is that access to electricity which is a proxy for socioeconomic status creates protective conditions such as access to ITNs and clean place of residence which reduce the likelihood of malaria (Dickinson et al., 2012, Worrall et al., 2003). A more direct plausible explanation is that the use of electrically operated equipment like fans within households with electricity can reduce mosquito bites. Nevertheless, literature on the association between access to electricity and malaria prevalence reports contrary findings in which access to electricity has been found to be positively associated with malaria prevalence (Tasciotti, 2017). Tasciotti (Tasciotti, 2017), for example, opined that access to electricity rather increases the malaria vector density in households which support the view that mosquitoes are attracted by light. This coupled with the fact that members in households with electricity are likely to spend more time in the evening outdoors increases their risk for mosquito bites (Tasciotti, 2017).

Our study also revealed that children in rural areas had a higher likelihood of testing positive for malaria. This finding supports the assumption that urbanization is protective against malaria in sub-Saharan Africa (Hay et al., 2005). Besides, our findings agree with the results of Afoakwah *et al* (Afoakwah et al., 2018) who reported rural children had a higher burden of malaria prevalence in Ghana. This may likely be explained by the fact that poverty is common in rural areas coupled with poor housing and environmental conditions that promote the breeding of mosquitoes. Our findings reflect the need to prioritise rural areas in malaria prevention policies.

We also found that having had dwelling areas sprayed against mosquito in the last twelve months before the survey was not protective against malaria prevalence. Yearly spraying appears not to offer much protection since mosquitoes breed virtually throughout the year in the environment, although the breeding rate and vector burden may be higher in the rainy season (Dery et al., 2010). On the contrary, some studies have reported a significant protective effect of household spraying when the effect was assessed at a shorter duration of 6 months (Afoakwah et al., 2018, Belete and Roro, 2016, Hamusse et al., 2012). For example, Belete & Roro (Belete and Roro, 2016) reported that spraying of the house environment in the last 6 months offers protection from malaria. Moreover, Hamusse *et al*. (Hamusse et al., 2012) showed that indoor residual spraying was effective in protecting against malaria within 6 months of the initial spraying. This underscores the need for continuous spraying at a shorter interval such as every 4–6 months to offer protection as yearly spraying appears not to be sufficient in preventing malaria.

The study found that compared to children in Western region (high rainforest ecological zone), their counterparts in the Greater Accra (Coastal Savannah), Ashanti (semi-deciduous rainforest), Northern (Guinea Savannah), Upper East (Sudan Savannah), and Upper West (Guinea Savannah) had a lower likelihood of malaria. This can be explained by the fact that that high rain forest ecological zone of the western region receives abundant rain compared to the other ecological zones. Rainfall is known to be associated with high densities of malaria vectors (Dery et al., 2010). With decrease rainfall in the other regions, children living there are less likely to have malaria compared to their counterparts in the high rainforest ecological zone of the western region.

### 5.1 Strengths and limitations

We have demonstrated the usefulness of machine learning techniques in predictive modelling for malaria in Ghana with an optimal level of sensitivities as seen in this study. The preliminary identification of variables for the final modelling using lasso, ridge and elastic net methods were less dependent on the researcher's intuition. The use of machine learning was also possible because a large nationality representative data was used. By using a nationally representative cross-sectional data, our findings can be generalized to children from other similar countries. Also, the use of big data approach to malaria modelling has additional benefits with regards to scalability and transferability to other settings with comparable data. Although our machine learning modelling appeared to have good predictive ability, the results are dependent on the data used in the development and validation. Larger datasets than the one we used would perhaps produce better-trained models. Finally, all associations observed in this study do not infer causality.

## 6 Conclusion

In summary, our study investigated the utility of machine learning approaches for predictive modelling of malaria prevalence among children under five years. The results showed evidence of concept and identified that age of the child, household wealth, place of residence, region of residence, anaemia status, and access to electricity was significantly predictive of malaria prevalence. The results (AU = 81.20%) show that the performance of our models is good at predicting under-five malaria prevalence. Beside identifying high-risk populations for cost-effective interventions, our study should serve as encouragement for malaria researchers in Ghana who are interested in machine learning and big data approaches in modelling malaria prevalence.

## CRediT authorship contribution statement

**Justice Moses K. Aheto:** Conceptualization, Methodology, Data curation, Formal analysis, Validation, Writing - original draft, Writing - review & editing, Project administration, Supervision. **Henry Ofori Duah:** Conceptualization, Writing - original draft, Writing - review & editing. **Pascal Agbadi:** Conceptualization, Methodology, Writing - original draft, Writing - review & editing. **Emmanuel Kweku Nakua:** Validation, Writing - original draft, Writing - review & editing, Supervision.

## Declaration of Competing Interest

The authors declare that they have no known competing financial interests or personal relationships that could have appeared to influence the work reported in this paper.
